# Supplementary material for: From sequence to enzyme mechanism using multi-label machine learning
Source: BMC Bioinformatics. 2014 May 19;15:150. doi: 10.1186/1471-2105-15-150 (PMC4229970; doi:10.1186/1471-2105-15-150)
Supplement: Additional file 2 — Java code of ml2db. Additional file ml2db_code.tar.gz contains the Java source code to run the multi-label machine learning experiments and save the results to database. The code’s Javadoc is included. [file 1471-2105-15-150-S2.zip › additional file 2/ml2db/utils/doc/overview-summary.html]

Overview


JavaScript is disabled on your browser.


- Overview
- Package
- Class
- Use
- Tree
- Deprecated
- Index
- Help

- Prev
- Next

- Frames
- No Frames

- All Classes

Packages

| Package | Description |
| --- | --- |
|  |  |
| --- | --- |
| cern.colt |  |
| cern.colt.function |  |
| cern.jet.random |  |
| cern.jet.random.engine |  |
| edu.cornell.lassp.houle.RngPack |  |
| uk.ac.standrews.utils.main |  |
| uk.ac.standrews.utils.main.collectionutils |  |
| uk.ac.standrews.utils.main.collectionutils.setutils |  |
| uk.ac.standrews.utils.main.database |  |
| uk.ac.standrews.utils.main.diff |  |
| uk.ac.standrews.utils.main.guiutils |  |
| uk.ac.standrews.utils.main.maputils |  |
| uk.ac.standrews.utils.main.setutils |  |
| uk.ac.standrews.utils.main.stats |  |
| uk.ac.standrews.utils.main.stringutils |  |
| uk.ac.standrews.utils.main.webutils |  |
| uk.ac.standrews.utils.main.webutils.simpledomparser |  |
| uk.ac.standrews.utils.test |  |
| uk.ac.standrews.utils.test.database |  |
| uk.ac.standrews.utils.test.maputils |  |
| uk.ac.standrews.utils.test.setutils |  |
| uk.ac.standrews.utils.test.stats |  |

- Overview
- Package
- Class
- Use
- Tree
- Deprecated
- Index
- Help

- Prev
- Next

- Frames
- No Frames

- All Classes
